# Supplementary figures and images for: Moderately low nitrogen application mitigate the negative effects of salt stress on annual ryegrass seedlings
Source: PeerJ. 2020 Dec 3;8:e10427. doi: 10.7717/peerj.10427 (PMC7719293; doi:10.7717/peerj.10427)

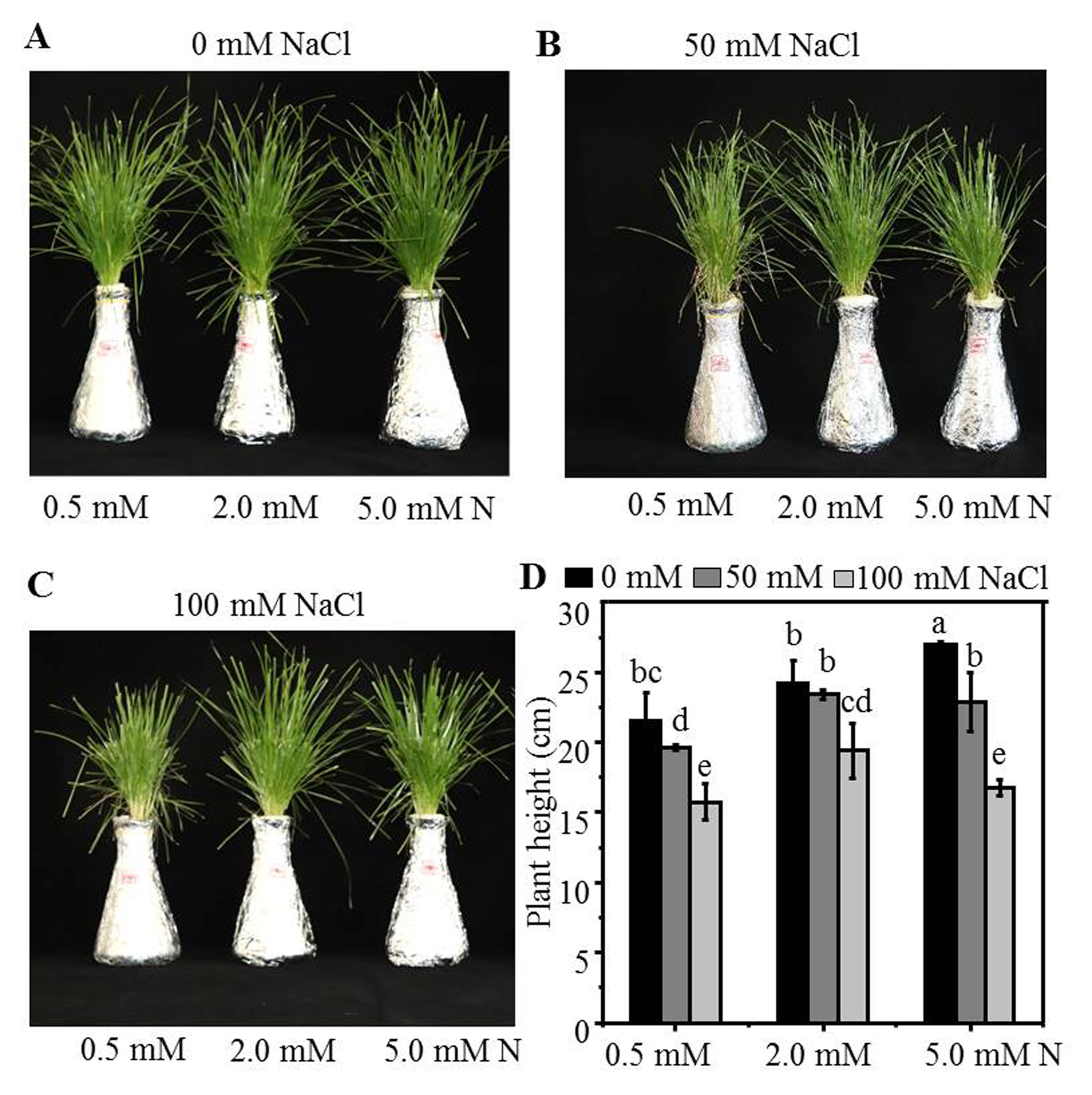

Supplement: Supplemental Information 1 — The seeds of annual ryegrass were cultured in soil for 20 days, and the seedlings cut to the same height were then transferred into different nitrogen level (0.5, 2.0, 5.0 mM) under 0 (A), 50 (B), 100 mM (C) NaCl stress in a hydroponic culture, and after being grown for 10 days, plant heights were measured. (D) Plant height at 10 days after transferred. Different letters above the columns indicate statistically significant differences at P ¡ 0.05 by Student’s t–test. [file peerj-08-10427-s001.jpg]

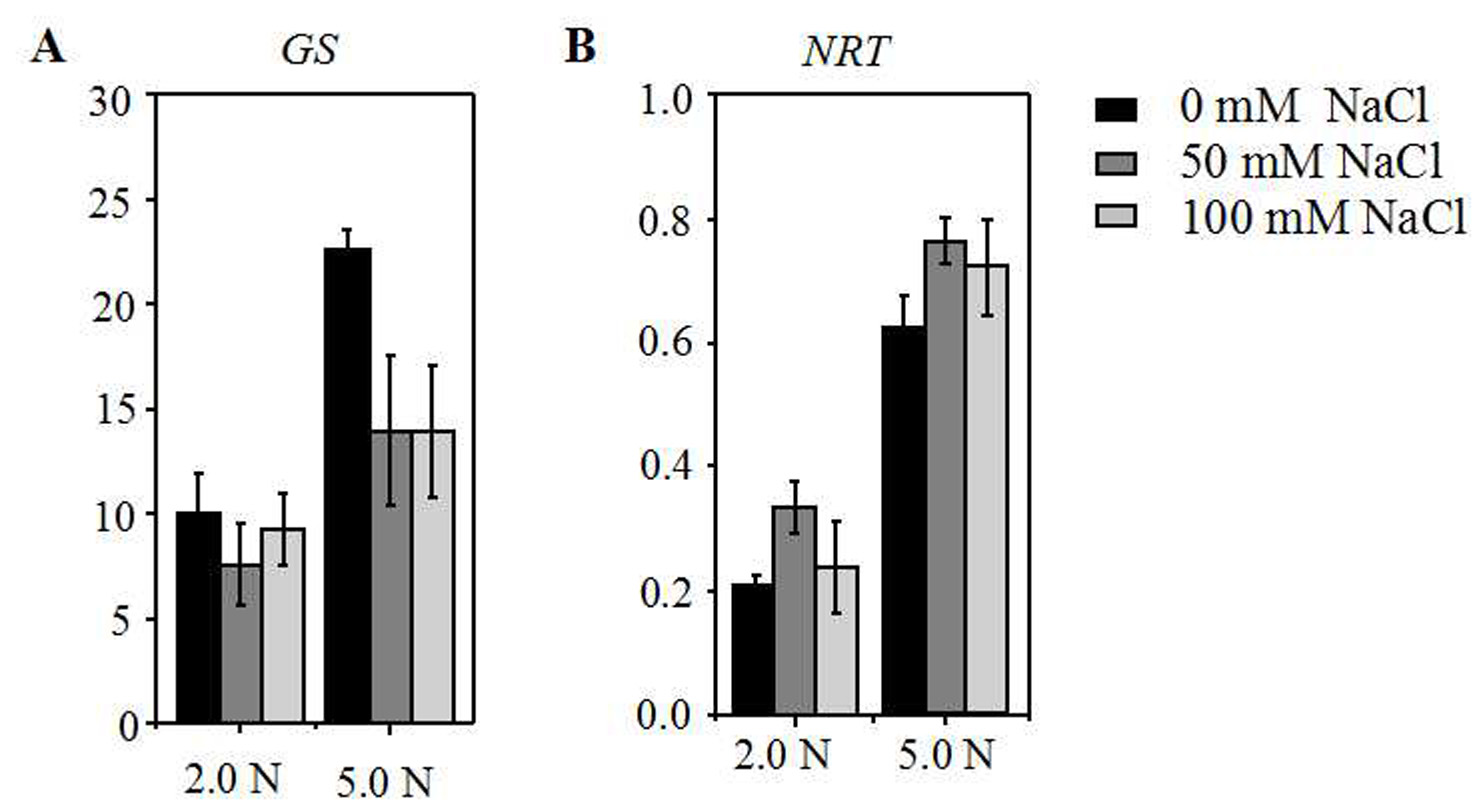

Supplement: Supplemental Information 2 — The annual ryegrass seedlings were grown under different nitrogen concentration (2.0, 5.0 mM) exposed to different salt stress (0, 50, 100 mM NaCl) respectively. Quantitative real-time PCR expression analysis of GS (A) and NRT (B) genes. Actin gene of ryegrass was used as an internal control. [file peerj-08-10427-s002.jpg]
