# Supplementary material for: Moderately low nitrogen application mitigate the negative effects of salt stress on annual ryegrass seedlings
Source: PeerJ. 2020 Dec 3;8:e10427. doi: 10.7717/peerj.10427 (PMC7719293; doi:10.7717/peerj.10427)
Supplement: Supplemental Information 3 [file peerj-08-10427-s003.docx]

Table S1. The primers used for quantitative real-time PCR.

| **primer name** | **primer sequence（5′-3′）** | **experiments** | |
| --- | --- | --- | --- |
| *q-GS-LP* | ACACCAAAGCGACAAAACCAA | expression analysis | |
| *q-GS-RP* | TCTCGGTTTAATGGGCTTTGA | expression analysis | |
| *q-NRT-LP* | TTGAGTACTGCGATGACGTGAA | expression analysis | |
| *q-NRT-RP* | CGGCGGTCCCTTGGTATC | expression analysis | |
| *q-Actin-LP* | CCGTTTTGTCGAGTTTGGT | expression analysis | |
| *q-Actin-RP* | AGCAACTGTAACCGAACATAGC | expression analysis | |
| *q-NR-LP* | ATGATCCGGGCGATGGA | expression analysis | |
| *q-NR-RP* | CAGGGAGTGCGCCTCCTT | | expression analysis |
